# Applying Quality Improvement Methodology to Standardize Pediatric Urinary Tract Infection Diagnosis and Management throughout a Healthcare System

**DOI:** 10.1097/pq9.0000000000000756

**Published:** 2024-08-21

**Authors:** Shannon H. Baumer-Mouradian, Lia C. Bradley, Sadia T. Ansari, Sri S. Chinta, Michelle L. Mitchell, Anika M. Nelson, Laura E. Marusinec, Kristine M. Wake, Karie A. Mantey, Ilanalee C. Cabrera, Jessica A. De Valk, Aaron P. Hanson, Elizabeth M. Witkowski, Glenn M. Bushee, Jonathan S. Ellison

**Affiliations:** * From the Department of Pediatrics, Medical College of Wisconsin, Children’s Corporate Center, Milwaukee, Wis.; † Quality Department, Children’s Wisconsin, Wauwatosa, Wis.; ‡ Urgent Care, Children’s Wisconsin, Wauwatosa, Wis.; § Children’s Medical Group, Children’s Wisconsin, Wauwatosa, Wis.; ¶ Office of Research Libraries, Medical College of Wisconsin, Milwaukee, Wis.; ‖ Department of Enterprise Safety, Children’s Wisconsin, Wauwatosa, Wis.; ** Department of Urology, Medical College of Wisconsin, Milwaukee, Wis.

## Abstract

**Background:**

Pediatric urinary tract infections (UTIs) require early diagnosis and appropriate treatment to avoid short- and long-term morbidity. Baseline data from 13,000 children across a regional health system demonstrated wide variation in UTI management, including antibiotic choice, duration, and dosing. In 2019, the local antibiotic stewardship team recommended cephalexin as the ideal first-line UTI treatment due to its effectiveness, narrow spectrum, low cost, and palatability. This project aimed to improve first-line prescription of cephalexin as an empiric antibiotic treatment for uncomplicated UTIs from 34% to 75% in children 60 days to 18 years of age presenting to any site within the healthcare system within 6 months.

**Methods:**

A multidisciplinary team of key stakeholders reviewed baseline data and developed three key drivers. These included a standardized UTI pathway, electronic health record enhancements, and provider education. Interventions were supported by a literature review and implemented via Plan-Do-Study-Act cycles with data monitored bimonthly. The primary outcome was the percentage of patients prescribed cephalexin for presumed UTI over the total number of presumed UTI diagnoses treated with empiric antibiotics throughout the healthcare system. The balancing measure included 14-day return visits for a UTI-related diagnosis across the system.

**Results:**

After the release of the updated UTI pathway, first-line cephalexin prescribing for UTI improved from 34% to 66%. There was no change in 14-day revisits for UTI.

**Conclusions:**

Standardizing the diagnosis and management of UTIs across the spectrum of coordinated care led to improved system-wide adherence to local antibiotic stewardship guidelines for empiric UTI treatment.

## INTRODUCTION

Urinary tract infections (UTIs) are a common cause of bacterial illness in childhood, affecting over one million (2.4%–2.8%) US children annually.^1^ Early diagnosis and appropriate treatment of UTIs are necessary to avoid short- and long-term morbidity, including sepsis, acute kidney injury, and renal scarring.^2^ However, overtreatment (ie, overly broad choice of empiric antibiotic) of UTIs increases the risk for antimicrobial resistance at both the patient and population levels.^3^ Therefore, there is a need for selective use of narrow-spectrum but effective antibiotics to treat confirmed UTIs or those with high clinical suspicion.

The American Academy of Pediatrics (AAP) 2011 UTI Guideline highlights the importance of selecting an antimicrobial agent based on local susceptibilities due to significant regional variability. Initial broad-spectrum antibiotic treatment poses the risk of future antibiotic resistance. Current data suggest that using empiric narrow-spectrum antibiotics to treat UTIs effectively avoids overtreatment.^4^ In 2019, our antibiotic stewardship team updated the local antibiogram to provide urine-specific susceptibility rates for common UTI pathogens, demonstrating high susceptibility (92%) to cephalexin and thus supporting it as a first-line treatment for uncomplicated UTI. After this change, however, wide variation in first-line antimicrobial treatment for UTI persisted across the emergency department (ED), urgent care (UC), and primary care domains within the integrated health system, highlighting an opportunity to align and improve care while promoting antimicrobial stewardship. Thus, we formed a system-wide UTI pathway committee at the request of the quality leadership team at the institution.

The global aim of this system-wide UTI pathway committee was to standardize the diagnosis, treatment, and follow-up care for patients presenting with signs and symptoms of UTI. The authors theorized that an evidence-based, system-wide pathway would improve consistency in empiric UTI treatment. The aim was to improve first-line prescription of cephalexin as an empiric antibiotic treatment for uncomplicated UTIs from 34% to 75% in children 3 months to younger than 18 years of age presenting to any site within the healthcare system within 6 months. We selected the target of 75% based on initial performance demonstrated by the ED, where the recommendation for cephalexin as a first-line antibiotic for UTIs was implemented and sustained at 74% since 2019, as well as targets reported in published literature on the success of UTI pathways at other pediatric institutions.^5^

## METHODS

### Local Context

#### Healthcare System

The integrated healthcare system comprises a 296-bed tertiary care, free-standing children’s hospital and level 1 pediatric trauma center/ED in Milwaukee, Wis., with seven regional UC and 24 children’s primary care clinics in Southeastern Wisconsin. A single electronic medical record (EMR) is shared across the healthcare system (EPIC, Verona, Wis.).

#### Antibiogram

The institutional antibiogram was reviewed in 2019, with data demonstrating high rates of susceptibility of *Escherichia coli*, *Klebsiella pneumoniae*, and *Proteus mirabilis* to cephalexin for treatment of uncomplicated UTI using adjusted mean inhibitory concentration breakpoints established by the Clinical and Laboratory Standards Institute in 2014 for these common uropathogens.^6^ Within this healthcare system, the adjusted urine breakpoint for cefazolin showed 92% susceptibility to *E. coli*, an increase from 77% susceptibility based on serum breakpoints.

#### Current State Assessment

Between January 1, 2020, and April 4, 2021, 8,840 children presented to the healthcare system with suspected UTI and 2,823 (32%) received empiric treatment. Suspected UTI was defined as the patient having a urinalysis with leukocyte esterase result (positive or negative) plus any UTI-related diagnosis (acute cystitis, cystitis, urinary tract infection, acute pyelonephritis, fever, dysuria, and abdominal pain). Before 2021, there was no institutional standardized approach to UTI diagnosis, empiric antibiotic choice/duration, or treatment failure. Instead, recommendations for first-line oral antibiotics for UTI varied by site: ED guidelines recommended cephalexin, UC guidelines recommended trimethoprim-sulfamethoxazole and primary care did not have a guideline. Additionally, parent experience surveys revealed dissatisfaction with inconsistent UTI care across the system.

Further review of baseline data demonstrated the great variability in empiric choice of antibiotics for uncomplicated UTIs, as reported in the Results section. Of note, initial, unfiltered data suggested ED providers prescribed cephalexin to 75% of suspected UTIs as a result of recommendations made by the antibiotic stewardship team in 2019. These recommendations had been incorporated into an ED-specific treatment guideline algorithm and disseminated through provider education in a practice culture that readily uses ED-specific guidelines for decision support. Further detailed chart review demonstrated the need to filter out additional diagnoses such as otitis media, pneumonia, and tonsillitis. New baseline ED data showed that 64% of providers treated UTIs with cephalexin; however, the team retained the 75% target for the specific aim.

### Background Assessment, Literature Review, and Development of a Key Driver Diagram

**Fig. 1.**
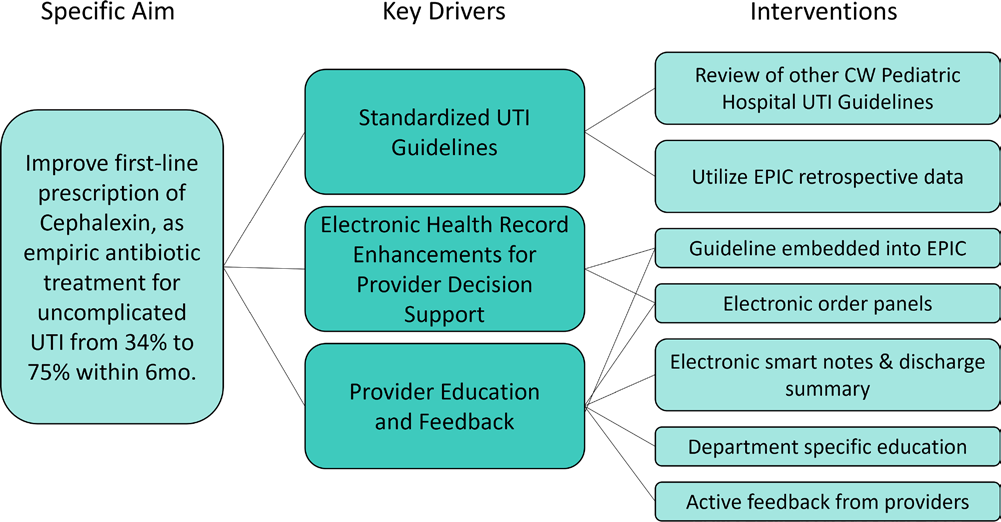
Key driver diagram for first-line antibiotic prescription for UTIs across an integrated health system.

The team mapped the process of UTI management across the system, reviewed existing local guidelines in each practice area, and performed a literature review. A literature review subcommittee, composed of nine physicians, a medical librarian, and a quality project lead, performed a comprehensive review of evidence addressing the questions “how to define UTI, who to test for UTI, how to test for UTI, choice and duration of antibiotics, failure of treatment, and when to image.” The literature review subcommittee systematically collated the literature review, publicly available pathways from other pediatric institutions, and local expert consensus. Based on these findings, the team formed a key driver diagram, which included a standardized UTI pathway, electronic health record enhancements, and provider education and feedback (Fig. 1).

### Interventions

#### Population

Children between the ages of 60 days and younger than 18 years who were evaluated in the healthcare system and treated for a UTI were included in the study. UTI treatment was defined as having an encounter where the child was prescribed antibiotics and had a UTI-related diagnosis (**Figure 1**, **Supplemental Digital Content 1,** http://links.lww.com/PQ9/A592). Children were excluded if they had known urologic conditions with a high risk for bacterial colonization or conditions where management of UTI would be potentially more complicated (ie, neurogenic bladder, vesicoureteral reflux, unresolved hydronephrosis, and nephrolithiasis), underwent genitourinary surgery within the last 30 days, had recurrent UTIs (≥3 per year), were on continuous antibiotic prophylaxis, were immunocompromised, or presented with concern for sepsis or pregnancy (**Figure 1**, **Supplemental Digital Content 1,** http://links.lww.com/PQ9/A592).

#### Standardized UTI Pathway

**Fig. 2.**
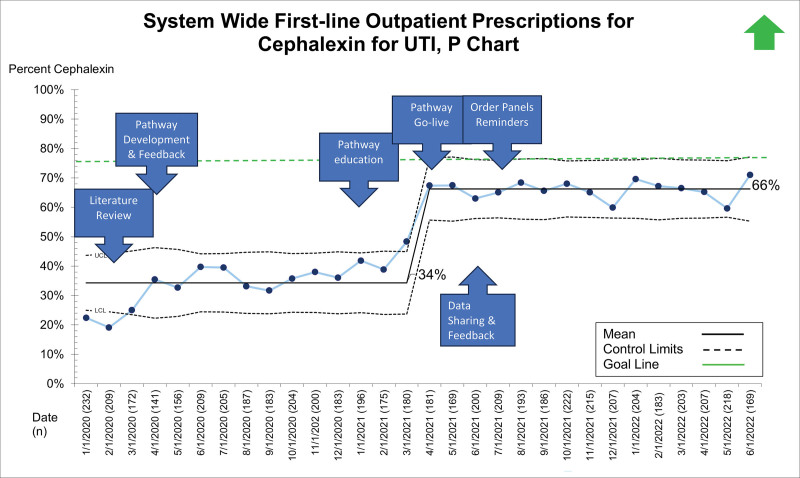
Interrupted time-series analysis of a system-wide pathway to improve cephalexin as a first-line antibiotic choice for UTI.

Following the assessment of the current state, a Plan-Do-Study-Act (PDSA) cycle was initiated to develop and disseminate a standardized system-wide pathway for UTI management. A multidisciplinary “UTI pathway team” was formed with 25 members providing representation from all practice areas within the healthcare system. An advanced practice nurse who served as the director of clinical pathways at the institution and two physicians with expertise in quality improvement led the team. Additional physician representation included providers from primary care, UC, ED, pediatric hospital medicine, urology, and infectious disease. Two physicians provided technical support with EMR provider builder training, a data analyst, and a librarian. This team created the system-wide UTI pathway encouraging empiric treatment with cephalexin based on specific UTI diagnosis codes (**Figure 2, Supplemental Digital Content 2,** http://links.lww.com/PQ9/A593). To ensure the pathway met the needs of each site, the team shared the pathway with the larger UTI pathway team in the spring of 2020 and underwent several iterations to ensure usability at all care sites.

#### EMR Enhancements

Modifications to the EMR included patient care note templates with clinical decision support, an order set, and patient discharge instructions. Clinical decision support included guidance on who to test and how to interpret urinalysis results. As microscopic urinalysis is not readily available at each clinical site, the team decided to include a reference to point-of-care urinalysis results for pathway decision-making.

The order set, which was created for UC and adapted for use in primary care, included a preselected prescription for cephalexin and a supporting statement to reinforce cephalexin as the preferred empiric antibiotic choice unless the patient had a cephalosporin allergy or severe allergy to penicillin. The antibiotic order automatically calculated the appropriate dose, frequency, and duration based on the patient’s weight and diagnosis (cystitis versus pyelonephritis), including a hard stop at the maximum recommended dose. The order set also included an option for trimethoprim-sulfamethoxazole for patients with drug allergy to cephalexin.

Standardized discharge instructions for all practice areas were developed. Instructions for empiric treatment included return precautions and signs of treatment failure, whereas instructions for watchful waiting described symptoms that may show the progression of an early UTI.

### Provider Education

Before UTI pathway implementation, the proposed pathway was presented to providers in each practice area to facilitate education and solicit feedback. We also presented the pathway and EMR tools to the pediatric residents at a house staff meeting. The UTI pathway, including EMR modifications such as order sets and discharge instructions, was published internally to the healthcare system’s intranet and activated as “Go Live” on April 5, 2021, where it was available to all providers and nurses. Following “Go Live,” pathway team members from various sites expressed the need for additional education; therefore, a second PDSA cycle focusing on pathway awareness and order set re-education was performed in October 2021 via staff and faculty meetings across UC, ED, and primary care.

### Feedback and Data Sharing with Clinicians

We built an automated data report to collect demographics and study measures throughout the healthcare system to monitor progress. Monthly emailed newsletters with control charts showing antibiotic prescription patterns for presumed UTIs were shared with the UTI pathway team. The control charts included data at the system level and data stratified for each practice area. Providers at all sites received quarterly data on antibiotic prescribing patterns specific to their site, with further clinic-specific prescribing shared at the discretion of UC and primary care representatives. Following monthly meetings during the first year of postpathway implementation, the UTI pathway team continued quarterly meetings to share data, review feedback, and discuss concerns related to the pathway from May 2021 to the present.

### Measures

The primary outcome was the percentage of patients prescribed cephalexin for presumed UTI over the total number of presumed UTI diagnoses treated with empiric antibiotics throughout the healthcare system. The balancing measure was the percent of return visits to any institutional clinical care site with a UTI-related diagnosis within 14 days of antibiotic prescription (**Figure 1, Supplemental Digital Content 1,** http://links.lww.com/PQ9/A592). The process measure was the EMR order set utilization rate in the primary care and UC settings.

### Analysis

Eligible patients (see Population) were pulled from our EMR based upon inclusion and exclusion of ICD-10 diagnosis codes, with exclusion diagnoses such as neurogenic bladder and nephrolithiasis filtered out during the data pull. Figure 1 (**Supplemental Digital Content 1,** http://links.lww.com/PQ9/A592) lists the inclusion and exclusion diagnoses. Descriptive statistics were used to describe the total pathway-eligible population. We used statistical process control charts to measure the impact of the interventions in real time. The centerline and control limits were revised when a special cause was noted as defined by eight consecutive points above or below the mean or a single data point above or below the upper or lower confidence interval.

#### Ethical Considerations

This study was determined to be a quality improvement project and thus exempt from local institutional review board review. The UTI risk stratification was adapted from the AAP Urinary Tract Infection Guideline (2011, 2016 revision); however, the UTI pathway team decided to remove race as a risk factor due to the evolving understanding of the role of racial inequality in healthcare and lack of a clear biological basis for race as a risk factor for UTI.^7,8^

## RESULTS

**Fig. 3.**
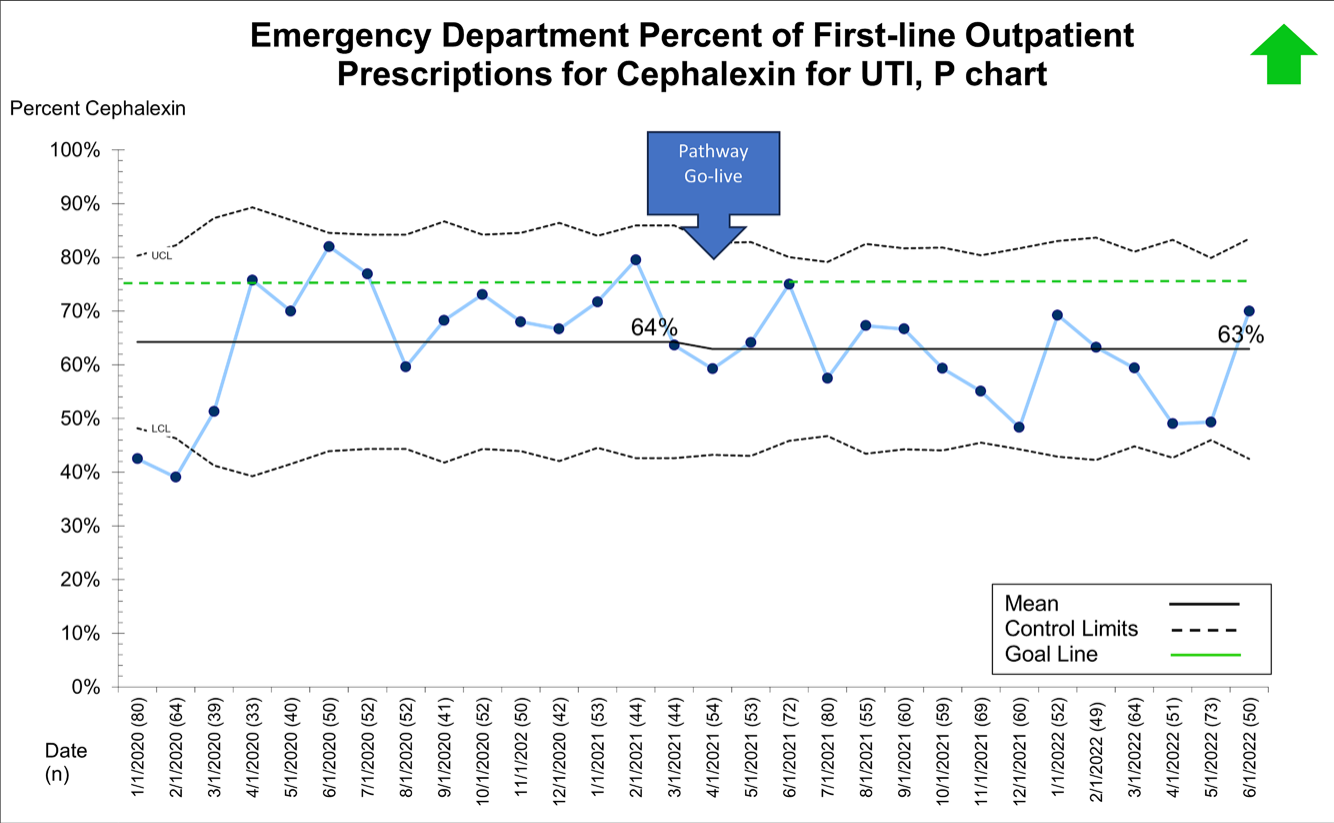
P chart demonstrating the percent of first-line prescriptions for cephalexin for outpatient pediatric UTIs within the ED.

**Fig. 4.**
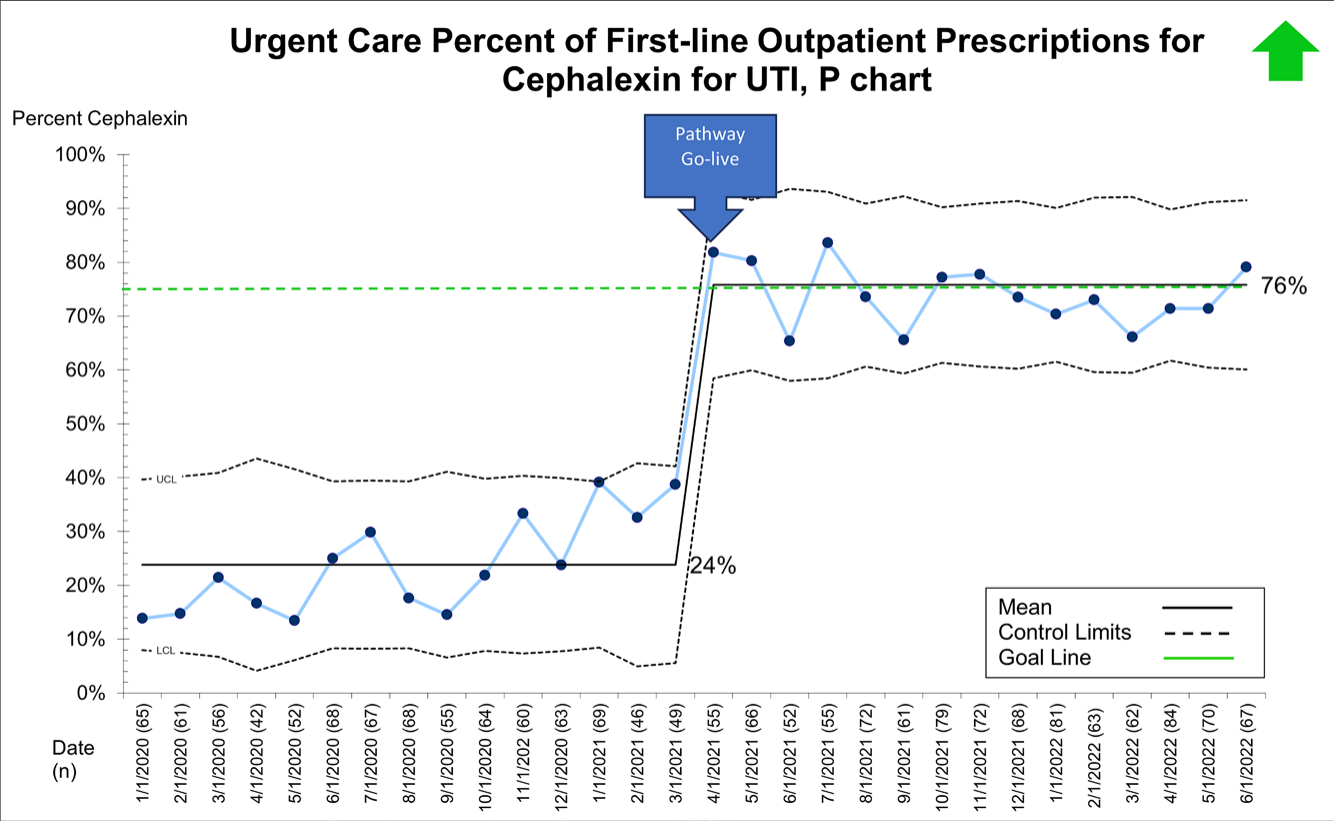
P chart demonstrating the percent of first-line prescriptions for cephalexin for outpatient pediatric UTIs within UC.

**Fig. 5.**
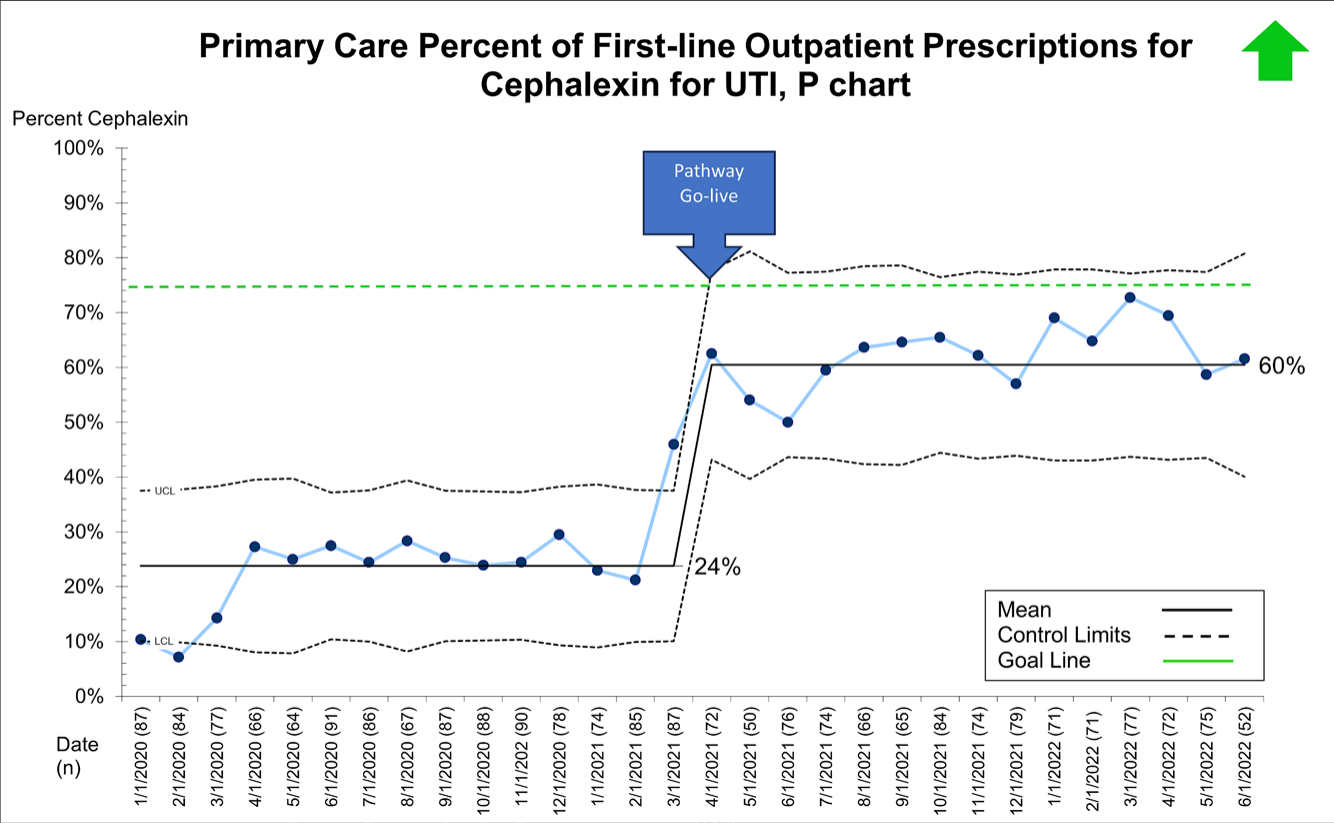
P chart demonstrating the percent of first-line prescriptions for cephalexin for outpatient pediatric UTIs within primary care.

Between January 1, 2020, and June 28, 2022, 19,363 patients presented to the ED, UC, and primary care sites with suspected UTI and received a urinalysis. Of these, 5,799 (29.9%) received empiric antibiotics for suspected UTI. The primary diagnoses for those receiving empiric antibiotics for suspected UTI included dysuria/urinary frequency, acute cystitis/pyelonephritis, fever, abdominal pain, constipation, vulvovaginitis, and viral illness. Patient demographics, stratified by presenting site, are displayed in Table 1. After the system-wide UTI pathway release on April 5, 2021, the first-line prescription of outpatient cephalexin for UTI across sites improved from 34% to 66% and was maintained for 14 months (Fig. 2). Cephalexin for empiric treatment of suspected UTI became the primary antibiotic of choice across the institution. Figures 3–5 display first-line antibiotic prescriptions across the study timeframe for each site separately (ie, ED, UC, and primary care). Prescribing improved from 24% to 60% in primary care and 24% to 76% in UC. ED prescriptions for cephalexin remained relatively stable at 64% versus 63%. Cephalexin prescribing for empiric treatment of suspected UTIs improved or was stable in all 19 primary care and all six UC clinics treating at least 10 UTIs during this timeframe. Five primary care clinics treated less than 10 patients for UTI, and only one improved cephalexin prescribing. Order set uptake was 58% in primary care and 54% in UC sites. There was no change in 14-day revisits (8%) for those patients treated with antibiotics for suspected UTI (**Figure 3, Supplemental Digital Content 3,** http://links.lww.com/PQ9/A594).

**Table 1.** Demographics of Patients Presenting to our Health System and Treated for a UTI from January 1, 2020, to June 28, 2022

	Primary Care	UC	ED	Total
Sex (N, %)				
Female	2,088 (92.0)	1,757 (92.8)	1,357 (82.9)	5,202
Male	181 (8.0)	136 (7.2)	280 (17.1)	597
Age (N, %)				
0 to <2	162 (7.1)	194 (10.2)	419 (25.6)	775
2 to <12	1,460 (64.3)	1,352 (71.4)	793 (48.4)	3,605
12 to <18	647 (28.5)	347 (18.3)	425 (26.0)	1,419
Insurance status (N, %)				
Commercial	1,574 (69.4)	1,262 (66.7)	478 (29.2)	3,314
Medicaid HMO	503 (22.2)	489 (25.8)	913 (55.8)	1,905
Medicaid	142 (6.3)	88 (4.6)	204 (12.5)	434
Medicare/other government	32 (1.4)	34 (1.8)	12 (0.7)	78
Unknown	18 (0.8)	20 (1.1)	30 (1.8)	68
SPS race/ethnicity (N, %)				
White	1,514 (66.7)	1,292 (6.8.3)	484 (29.6)	3,290
Black or African American	326 (14.4)	197 (10.4)	562 (34.3)	1,085
Multiracial, Hispanic	224 (9.9)	242 (12.8)	426 (26.0)	892
Unknown	80 (3.5)	69 (3.6)	20 (1.2)	169
Multiracial, Non-Hispanic	50 (2.2)	46 (2.4)	46 (2.8)	142
Asian	46 (2.0)	22 (1.2)	57 (3.5)	125
Hispanic or Latino or Latinx	22 (0.3)	20 (0.2)	35 (0.3)	77
American Indian or Alaska Native	6 (0.0)	3 (0.1)	5 (0.1)	14
Native Hawaiian or Other Pacific Islander	0 (0.0)	2 (0.1)	1 (0.1)	3
Multiracial, unknown ethnicity	1 (0.1)	0 (0.0)	1 (0.1)	2

CMG, Primary Care Children’s Medical Group; HMO, health maintenance organization.

## DISCUSSION

### Summary

This project highlights how a multidisciplinary collaboration of leadership across the healthcare system can successfully optimize medical management and provide a consistent and sustainable standard of care across an integrated healthcare system. An interdisciplinary team successfully standardized the evaluation and treatment of children with UTIs throughout a regional healthcare system, as evidenced by improved adherence to the locally recommended first-line antibiotic choice of cephalexin without harming treatment failures.

### Interpretation

Our pathway’s success depended on our ability to incorporate local context, collaborate in a multidisciplinary fashion, and leverage an integrated health model. We learned several key lessons during this process. First, incorporating local context into planning a multisite quality improvement effort was vital to understanding the most appropriate UTI testing and treatment approach. For example, microscopic urinalysis was unavailable at all primary and UC sites and could add substantial time to evaluations depending on laboratory availability. As such, we created diagnostic recommendations that were broad-based and applicable across all participating sites. Second, although standardization of pathway recommendations across all sites was vital to success, the implementation strategies at each site varied due to site-specific workflows and microcultures, such as the use of order sets. The relationship between process measures, such as order set utilization, and outcome measures was strong in UC and primary care sites. Our experience reflected that of others who have shown effective change-management strategies for antibiotic choice in pediatric UTI by leveraging clinical decision support.^9^ However, as ED workflows did not support order set utilization for any diagnosis at the time of pathway implementation, we felt that focusing on order set utilization across all sites (including ED) would have detracted from the successful goal of antibiotic prescription patterns. Kline et al^10^ reported similar success with implementing evidence-based treatment recommendations for pediatric UTI within their ED, primarily leveraging education and provider feedback. Finally, by incorporating acute and primary care sites into a single data collection system, the UTI team monitored individual site-level progress, provided site-specific feedback, tracked system-level performance, and monitored the balancing measure of 14-day return visits for UTIs.

Previous UTI-standardized pathways in acute and outpatient settings have improved prescribing patterns and sustainably reduced care variation. Adherence to the first-line antibiotic choice described in our work is comparable to adherence reported in other experiences.^5,11^ However, neither of these previously published efforts looked to standardize efforts across the entire health system as this project was able to do.

Finally, ED-prescribing patterns of cephalexin for first-line treatment of uncomplicated UTI did not improve in the manner seen in primary care and UC. This observation is likely because the UTI pathway team focused heavily on interventions in primary care and UC to facilitate a significant change in practice to offer cephalexin as a first-line treatment. Additional ED obstacles included competing priorities due to high patient volumes and critically ill patients and frequent turnover of ED trainees from multiple institutions, which introduce challenges to trainee education. In the future, the team plans to identify successful interventions applied in UC and primary care, such as discharge order sets, and determine if similar interventions could further improve cephalexin prescribing patterns in the ED.

This project has several limitations worth mentioning. Other institutions seeking to replicate this project should base empiric antibiotic recommendations on their local antibiotic susceptibility patterns. Additionally, despite team efforts to review the relevant literature, AAP guidelines, and national pediatric UTI management pathways, expert consensus was needed when data were lacking.^12–14^ The impact of a system-wide shift to first-generation cephalosporins on local antibiotic resistance patterns has not been well-studied. A longer follow-up is needed to determine if cephalexin is the best empiric antibiotic choice based on susceptibility patterns. However, Poole et al^5^ found no difference in the proportion of urinary isolates resistant to the prescription of choice for up to 4 years following the institution of their clinical UTI pathway. Data presented here may not precisely reflect the granular and clinician-driven inclusion and exclusion criteria for the pathway since certain criteria may not be represented in the ICD-10 billing data used to create the analytic cohort. Additionally, prescriptions for febrile non-UTI-related diagnoses such as acute otitis media, pneumonia, or streptococcus pharyngitis may be included in the dataset, which would be biased against the primary aim. Nonetheless, our ICD-10 inclusion and exclusion codes were consistent throughout implementation and PDSA cycles and likely reflected the most targeted patients for the system-wide pathway. Additionally, opportunities exist to investigate antibiotic stewardship with the expansion of data collection. For instance, the primary analysis did not include antibiotic dose, frequency, and duration as data infrastructure did not initially support their collection.

### Concluding Summary

This work presents an opportunity to improve the management of pediatric UTIs by integrating primary and acute care sites under the guidance of a single pathway. It has highlighted several strategies for integrated care pathways that may be applied to any disease entity. By structuring pathways that utilize standard available resources (ie, availability of urinalysis tests) and accommodating a flexible dissemination structure, standardized work in an integrated health system is impactful and sustainable.

## ACKNOWLEDGMENT

This work would not have been possible without the administrative support and leadership of the following individuals: Karie Mantey, MD, Sarah Bauer, MD, Ben Landgraf, MD, Jennifer Miller, PA-C, Jaspreet Samra, Danielle Smith, MSN, RN, CNL, Katie Ray PharmD, Ian Reineking, and Robert Rohloff, MD.

## Supplementary Material


