# Supplementary material for: Applying Quality Improvement Methodology to Standardize Pediatric Urinary Tract Infection Diagnosis and Management throughout a Healthcare System
Source: Pediatr Qual Saf. 2024 Aug 21;9(5):e756. doi: 10.1097/pq9.0000000000000756 (PMC11338250; doi:10.1097/pq9.0000000000000756)
Supplement: Supplementary file 1 [file pqs-9-e756-s001.pdf]

URINARY TRACT INFECTION

Outpatient Testing and Empiric Treatment:

Intended for patients age >60 days to <18 years with presumed or definite UTI

Exclusion Criteria:
- Urinary tract instrumentation or catheter (incl. intermittent cath)
- Known urologic conditions (neurogenic bladder, current hydronephrosis, posterior urethral valves, known/concern for nephrolithiasis, vesicoureteral reflux)
- Recent (≤30 days) GU surgery
- Recurrent UTIs (≥3/year)
- On antibiotic prophylaxis for UTI
- Immunocompromised states
- Concern for sepsis
- Pregnancy

Signs/Symptoms of UTI

Age >60 days to <2 years (not toilet trained): Test based on risk for UTI (Option to use UTICalc* https://uticalc.pitt.edu/)

*If using UTICalc, consider selecting non-black for all patients, to reduce the potential risk for testing bias

Age <6 months: Obtain a catheterized specimen for UA and urine culture

Age ≥6 months to <2 years – 2 options:
1) Obtain cath specimen for UA and urine culture **OR**
2) Obtain bagged specimen for UA. If +nitrite or ≥trace leukocyte esterase on UA, obtain **cath** specimen for urine culture

Additional testing considerations for sexually active adolescents

Clean Void Urine Specimen Collection teaching sheets

Age ≥2 years (toilet-trained): Obtain clean catch UA (If unable to obtain clean catch, refer to testing for ≥6 mos to <2 yrs)

Review UA results for leukocyte esterase (LE) and nitrite

Decision aid for nitrite negative UAs

Positive nitrite **and/or** large (3+) LE:
- Send urine culture
- Start empiric antibiotics

Negative nitrite **and** moderate (2+), small (1+), or trace LE:
- Send urine culture based on clinical judgment
- If sending a urine culture, use clinical judgment to either start empiric antibiotics or wait for culture results – consider waiting if trace or small (1+) LE

Negative nitrite **and** negative LE:
- Do not send urine culture unless obtained via catheterization *or* high clinical suspicion for UTI
- Do not start empiric antibiotics
- Consider alternative diagnosis

Treatment Failure definition

Does the patient require escalation of care for suspected UTI? Toxic-appearance, dehydration, inability to tolerate oral/enteral fluids or antibiotics, concern for failure of outpatient management

Yes → Prepare patient/family for ED visit or admission

Hospital Medicine CPG for Febrile UTI in Young Children

No → Prepare patient for discharge. If indicated, prescribe empiric antibiotics

Outpatient Empiric Antibiotic Recommendations *^ — Cystitis vs Pyelonephritis — Cephalexin for UTI

Age >60 days to <2 years	Age 2 to <12 years	Age ≥12 years
Cystitis/Pyelonephritis: cephalexin for 10 days 25 mg/kg/dose TID	Cystitis: cephalexin for 7 days 25 mg/kg/dose [max 500 mg/dose] TID<br>Pyelonephritis: cephalexin for 10 days 25 mg/kg/dose [max 1000 mg/dose] TID	Cystitis: cephalexin for 3 days 25 mg/kg/dose [max 500 mg/dose] TID<br>Pyelonephritis: cephalexin for 10 days 25 mg/kg/dose [1000 mg/dose] TID

*If Hx of prior UTI, select empiric therapy based on previous urine culture sensitivities

^If allergy to cephalosporins or severe IgE-mediated reaction (i.e. anaphylaxis or anaphylactoid reaction) to penicillins (incl. amoxicillin), consider trimethoprim/sulfamethoxazole 5-6 mg/kg/dose [max 160 mg/dose trimethoprim for cystitis or pyelonephritis] BID for the duration recommended for cephalexin based on age and diagnosis

Interpreting Urine Culture Results

Clinical Follow-Up and Imaging Recommendations

Children's Wisconsin

For questions concerning this work, contact UTIPathway@chw.org
© 2018 Children's Hospital and Health System, Inc.
See Medical Disclaimer

Updated: March, 2021
Valid until: March, 2026

Interpreting Urine Culture Results

Definition of a UTI:
Clinical signs and symptoms of UTI and/or abnormal UA (positive leukocyte esterase and/or nitrite) **AND** growth of a urinary pathogen* at or above the diagnostic threshold

SPECIMEN SOURCE	**DEFINITE UTI** (cfu/mL)	**POSSIBLE UTI** (cfu/mL)
Catheterization	≥50,000 cfu/mL	≥10,000 cfu/mL
Clean-catch	≥100,000 cfu/mL	≥50,000 cfu/mL

*A positive urine culture may include more than one pathogen, as long as a urinary pathogen is present at or above the required threshold

Urinary Pathogens:
- *Citrobacter sp.*
- *Corynebacterium urealyticum*
- *Enterobacter sp.*
- *Enterococcus sp.*
- *E. Coli*
- *Klebsiella sp.*
- *Morganella morganii*
- *Proteus sp.*
- *Pseudomonas sp.*
- *Serratia sp.*
- *Staphylococcus aureus*
- *Streptococcus agalactiae* group B

Common Contaminants*:
- *Aerococcus sp.*
- *Corynebacterium sp.*
- *Coryneform bacteria*
- *Lactobacillus sp.*
- Coagulase-negative staphylococci (incl. *S. epidermidis, S. simulans*)
- Alpha-hemolytic streptococci (incl. *S. viridans, S. pneumoniae*)

*Contaminants should not be treated at any level of growth

Urine Culture Follow-Up

See Treatment Failure | See Tests of Cure

UA RESULTS	CULTURE RESULTS	RECOMMENDATIONS
UA positive and started on empiric antibiotics	Cfu criteria met for **definite or possible** UTI	• Check sensitivities, change antibiotic if necessary*<br>• See imaging and follow-up recommendations
	Contaminant or negative	• Stop treatment<br>• Inform family that child did not have UTI
UA positive and not started on empiric antibiotics	Cfu criteria met for **definite** UTI	• Check sensitivities, start on appropriate antibiotic<br>• See imaging and follow-up recommendations
	Cfu criteria met for **possible** UTI	• Check patient:<br>• If febrile and/or persistent symptoms:<br>• Check sensitivities, start on appropriate antibiotic therapy<br>• See imaging and follow-up recommendations<br>• If afebrile and Sx improving/resolved:<br>• No treatment<br>• Inform family that child did not have UTI
	Contaminant or negative	• No treatment<br>• Inform family that child did not have UTI
UA negative	Cfu criteria met for **definite or possible** UTI	• Check patient:<br>• If febrile and/or persistent symptoms:<br>• Consider repeating the urine culture **or** starting on appropriate antibiotic therapy based on sensitivities<br>• See imaging and follow-up recommendations<br>• If afebrile and Sx improving/resolved:<br>• No treatment<br>• Inform family that child did not have UTI
	Contaminant or negative	• No treatment<br>• Inform family that child did not have UTI

*If clinical symptoms are improving, it is not necessary to change antibiotics, even if culture sensitivities show resistance

Outpatient Testing and Empiric Treatment   Clinical Follow-Up and Imaging Recommendations

© 2018 Children's Hospital and Health System, Inc.
See Medical Disclaimer

Clinical Follow-Up and Imaging Recommendations

- After diagnosis with febrile UTI/pyelonephritis, patients and families should receive education about the importance of seeking prompt medical evaluation (within 48 hours) for future febrile illnesses

Imaging Recommendations

- Goal of imaging in febrile UTI/pyelonephritis: to identify patients with vesicoureteral reflux (VUR) and to rule out the small percentage (~1%) of patients with structural anomalies of the urinary tract
- Imaging is not typically indicated for recurrent, non-febrile UTIs, unless there are other symptoms (i.e. gross hematuria or recurrent flank pain) or the patient has recurrent (≥3/year) UTIs with the same organism(s) concerning for nidus, such as stone

When to Obtain a Renal and Bladder Ultrasound (RBUS)

Age >60 days to <2 years

- RBUS after:
  - 1st febrile UTI/ pyelonephritis

Timing of RBUS:

- If hospitalized and no improvement after 48 hours on appropriate therapy:
  - Obtain during acute phase of illness
- For all other patients:
  - Wait at least 30 days to obtain RBUS

Age ≥2 years

- RBUS after:
  - 2nd febrile UTI/pyelonephritis
  - Non-*E. Coli* febrile UTI/pyelonephritis
  - Febrile UTI/pyelonephritis in a patient who has a first-degree relative with VUR

When to Obtain a Voiding Cystourethrogram (VCUG)

Age >60 days to ≤18 years

- VCUG after:
  - 2nd febrile UTI/pyelonephritis
  - Abnormal RBUS*
  - 1st febrile UTI/pyelonephritis plus any of the following:
    - Non-*E. Coli* UTI
    - Parent or sibling with VUR
    - High provider index of suspicion for clinically significant VUR, including severe presentation of febrile UTI (i.e. prolonged or complicated admission) or multi-drug resistant organism
    - Parental concern and desire to evaluate for VUR

Timing of VCUG:

- VCUG can be safely performed once the patient is afebrile and has stabilized from the infection

Urology Referral Recommendations (all ages)

*Abnormal RBUS/Imaging:
Moderate to severe hydronephrosis/pelviectasis, Hydroureter, Ureteral duplication, Evidence of renal scarring

- A Referral to Urology should be placed for:
  - Boys after the 1st febrile UTI/pyelonephritis, irrespective of imaging results
  - Girls after the 1st febrile UTI/pyelonephritis, with abnormal imaging*
  - Boys and Girls after the 2nd febrile UTI/pyelonephritis, irrespective of imaging results
  - Consider an outpatient referral for boys or girls after the 1st febrile UTI/pyelonephritis, if UTI required inpatient evaluation
  - Desire by the family or the primary provider to seek specialist evaluation following the 1st febrile UTI/pyelonephritis
- The Urology Consult can be performed as an e-consult or in-person consult, based on family and provider preferences

Outpatient Testing and Empiric Treatment Interpreting Urine Culture Results

© 2018 Children's Hospital and Health System, Inc.
See Medical Disclaimer

Appendix: UTI Risk Stratification: Age >60 days to <2 years

**Female Risk Factors:**
• Age < 12 months
• Temperature ≥ 39 C
• Fever ≥ 48 hours
• No other source of infection
**1 risk factor** (≤ 1% risk) = LOW risk: do not test unless high clinical suspicion for UTI
**2 risk factors** (≤ 2% risk) = INTERMEDIATE risk: consider testing based on clinical assessment
**3+ risk factors** (≥ 2% risk) = HIGH risk: testing is recommended

**Male Risk Factors:**
• Uncircumcised (= 2 risk factors)
• Temperature ≥ 39 C
• Fever ≥ 24 hours
• No other source of infection
**1 risk factor** (≤ 1% risk) = LOW risk: do not test unless high clinical suspicion for UTI
**2 risk factors** (≤ 2% risk) = INTERMEDIATE risk: consider testing based on clinical assessment
**3+ risk factors** (≥ 2% risk) = HIGH risk: testing is recommended

Adapted from the American Academy of Pediatrics Urinary Tract Infection Guideline (2011, 2016 revision) with modification. The UTI Pathway team made the decision to remove race as a risk factor, due to the evolving understanding of the role of racial inequality in healthcare and lack of a clear biological basis for race as a risk factor for UTI (Kowalsky et al, 2020; Vyas et al, 2020). In this context, utilizing race as a factor in the clinical decision-tools risks perpetuating the same inequalities that generated these data in the first place. The team acknowledges the utility of UTICalc as a tool to aid provider decision-making but recommends that non-black be selected for all patients, in order to reduce the potential risk for racial bias.

Return to: Outpatient Testing and Empiric Treatment

© 2018 Children's Hospital and Health System, Inc.
See Medical Disclaimer

Appendix: CW Clean Void Urine Specimen teaching sheets

- Clean Void Urine Specimen: Girls (English)
- Clean Void Urine Specimen: Girls (Spanish)
- Clean Void Urine Specimen: Boys (English)
- Clean Void Urine Specimen: Boys (Spanish)

Return to: Outpatient Testing and Empiric Treatment

Appendix: Additional Testing Considerations for Sexually Active Adolescents

- For males: obtain first void ('dirty') urine specimen for Gonococcus (GC)/Chlamydia (Chl) testing
- For females: obtain vaginal self-swab or first void urine specimen for Gonococcus (GC)/Chlamydia (Chl) testing; consider pregnancy testing
- Obtain clean void specimen for UA +/- Urine Culture
- For males and females: Consider HSV testing if visible lesions; consider Syphilis Screen if GC/Chl positive

Return to: Outpatient Testing and Empiric Treatment

Appendix: Leukocyte Esterase vs Leukocytes

- The value that CW lab reports as 'Leukocytes' is a direct reference to 'Leukocyte esterase'

Return to: Outpatient Testing and Empiric Treatment

© 2018 Children's Hospital and Health System, Inc.
See Medical Disclaimer

Decision Aid for Nitrite Negative UAs:

- Purpose: to assist provider decision-making for patients with nitrite negative UAs
- Developed through a review of 2 years of data (1/1/2019 – 12/31/2020) from patients with a negative nitrite UA and paired urine culture who were seen at Children's Wisconsin Primary Care, Urgent Care, or Emergency Department
- Positive urine culture defined as growth of ≥50,000 CFU/mL of known uropathogen for a clean void urine specimen and ≥10,000 CFU/mL of a known uropathogen for a catheterized urine specimen (*combining the pathway definitions for possible and definite UTI)

Catheterized (n = 850)

Leukocyte esterase concentration		% with positive urine culture
Negative	(n = 628)	2.6
Trace	(n = 65)	18.5
Small (1+)	(n = 59)	54.2
Moderate (2+)	(n = 56)	69.6
Large (3+)	(n = 42)	78.6

Clean Void: ages 2-11 (n = 4231)

Leukocyte esterase concentration		% with positive urine culture
Negative	(n = 1431)	3.2
Trace	(n = 886)	8.5
Small (1+)	(n = 835)	17.4
Moderate (2+)	(n = 762)	27.2
Large (3+)	(n = 317)	40.7

Clean Void: ages 12-18 (n = 1138)

Leukocyte esterase concentration		% with positive urine culture
Negative	(n = 396)	4.0
Trace	(n = 222)	15.8
Small (1+)	(n = 250)	32.4
Moderate (2+)	(n = 186)	34.4
Large (3+)	(n = 84)	50.0

Return to: Outpatient Testing and Empiric Treatment

© 2018 Children's Hospital and Health System, Inc.
See Medical Disclaimer

Appendix: Clinical Differentiation between Cystitis and Pyelonephritis

- Clinical Signs/Symptoms that are suggestive of a urinary tract infection:
    - Unexplained fever (>38° C)
    - Dysuria
    - Increased urgency
    - Increased frequency
    - Abnormal urinalysis (+Nitrite, LE present)
- Consider a diagnosis of pyelonephritis for:
    - All children age< 2 years of age with fever and urinary symptoms. (Children <2 years with fever and urinary symptoms are considered to have presumptive pyelonephritis.)
    - Older children with any of the following symptoms:
        - Fever/chills
        - Flank pain
        - Nausea/Vomiting

Return to: Outpatient Testing and Empiric Treatment

© 2018 Children's Hospital and Health System, Inc.
See Medical Disclaimer

Appendix: Cephalexin for UTI Rationale

In assessing our local antibiogram using CLSI urine specific breakpoints, the chosen concentrations at which bacteria are considered susceptible or resistant to a specific antibiotic, the antimicrobial stewardship program determined cephalexin is the narrowest spectrum antibiotic that could empirically cover the majority of likely pathogens. Although cefdinir has frequently been prescribed to treat outpatient UTIs in our system, it is unnecessarily broad for the treatment of common urinary pathogens (Table 1). Additionally, the pharmacokinetic profile of cefdinir is inferior to cephalexin. Cephalexin has significantly higher bioavailability and less protein binding than cefdinir, though does require a more frequent dosing schedule due to its short half-life (Table 2). Although trimethoprim-sulfamethoxazole is another commonly used agent for outpatient UTI treatment, our antibiogram reveals lower coverage for *Escherichia coli* compared to cephalosporins when accounting for urine specific breakpoints.

The Clinical and Laboratory Standards Institute (CLSI) created urine specific breakpoints for enterobacteriaceae in 2014.[1] These breakpoints predict susceptibility for cefazolin for the most common urine pathogens (*Escherichia coli, Klebsiella Pneumoniae,* and *Proteus mirabilis*) at a higher minimum inhibitory concentration (MIC) than non-urine specimens. Furthermore, cefazolin may be used as a surrogate to predict susceptibility to other cephalosporin antibiotics (i.e., cefaclor, cefdinir, cefpodoxime, cefprozil, cefuroxime, cephalexin, and loracarbef) (Table 1).

Table 1. Percent of susceptible isolates for common urinary pathogens among pediatric outpatients in our health system

	***Escherichia coli* (n = 625)**	***Klebsiella Pneumoniae* (n = 45)**	***Proteus mirabilis* (n = 51)**
	Percent susceptible		
**Ampicillin/sulbactam**	57	85	94
**Cefazolin***	91	95	94
**Ceftriaxone****	93	98	98
**Sulfamethoxazole/trimethoprim**	77	93	92

* Using the CLSI urine-specific breakpoints which can be used as a surrogate to predict susceptibility to other cephalosporin antibiotics (i.e., cefaclor, cefdinir, cefpodoxime, cefprozil, cefuroxime, cephalexin, and loracarbef)
** Ceftriaxone breakpoints cannot be directly used to predict susceptibility to cefdinir. There are cefdinir specific breakpoints for Enterobacteriaceae, but it is not on the CHW susceptibility panel. When cefazolin is used as a surrogate for oral cephalosporins and interpreted using the uncomplicated UTI breakpoints for E. coli, Kleb pneumo and P. mirabilis, cefdinir resistance may be overcalled. Cefdinir may also be susceptible when cefazolin is reported as resistant.

Table 2. Comparison of pharmacokinetic profiles of cefdinir and cephalexin

© 2018 Children's Hospital and Health System, Inc.
See Medical Disclaimer

	**Cefdinir***	**Cephalexin^**
Oral bioavailability (%)	25	90
Peak serum concentration (µg/mL)	1.6	18
Range of urine concentration (µg/mL)	21 – 139	5,000 – 10,000
Protein binding (%)	60 – 70	5 – 15
Half-life (hours)	1.7	1 – 2

Adapted from Gilbert 2015[2] and Gilbert 2006[3]
* Based on a single 300 mg dose
^ All data based on a single 500 mg dose, except urine drug concentration, which is based on a single 1,000 mg dose

References:

1. Clinical and Laboratory Standards Institute (CLSI). Performance standards for antimicrobial susceptibility testing-fourth informational supplement (Update). CLSI document. Wayne: CLSI; 2014.

2. Gilbert DN, Eliopoulos GM, Chambers HF, Saag MS, et al. *The Sanford guide to antimicrobial therapy 2015.* Sperryville, VA: Antimicrobial Therapy, Inc.

3. Gilbert DN. Urinary Tract Infections in Patients with Chronic Renal Insufficiency. *Clin J Am Soc Nephrol*. 2006;1:327-331.

Return to: Outpatient Testing and Empiric Treatment

© 2018 Children's Hospital and Health System, Inc.
See Medical Disclaimer

Appendix: Treatment Failure

- Failed outpatient therapy as defined by persistent clinical symptoms or lack of meaningful clinical improvement beyond 48 hours on appropriate antimicrobial therapy
- In the event of treatment failure, consider:
    - Resistant organism?
    - Poor adherence to treatment (i.e. reticent to take meds, dosing able schedule)?
    - Poor PO intake or emesis leading to poor drug absorption?
    - Source control (i.e. urinary obstruction or abscess), if still febrile?
    - Alternate diagnosis (i.e. constipation), if dysuria persists on correct treatment?

Return to: Outpatient Testing and Empiric Treatment

Return to: Interpreting Urine Culture Results

Appendix: Tests of Cure

- Tests of cure are NOT recommended
- The AAP Section on Nephrology and the American Society of Pediatric Nephrology has issued the following statement regarding tests of cure for pediatric patients with UTIs:

  > "Avoid ordering follow-up urine culure after treatment for an uncomplicated urinary tract infection (UTI) in patients that show evidence of clinical resolution of infection. Studies have shown that clinical resolution of infection is adequate for determining effectiveness of antibiotic therapy after treatment for UTI." (AAP Section on Nephrology & American Society of Pediatric Nephrology, 2018)

Return to: Interpreting Urine Culture Results

© 2018 Children's Hospital and Health System, Inc.
See Medical Disclaimer

References:

American Academy of Pediatrics Section on Nephrology, American Society of Pediatric Nephrology. Follow-up urine cultures after treatment for uncomplicated UTI. 2018 July. Available at: https://www.choosingwisely.org/clinician-lists/aap-aspn-follow-up-urine-cultures-after-treatment-for-uncomplicated-uti/. Accessed April 16, 2020.

Children's Hospital Colorado, Mistry R, Gaensbauer J, et al. 2017 April. Urinary Tract Infection (UTI). Available from: https://www.childrenscolorado.org/globalassets/healthcare-professionals/clinical-pathways/urinary-tract-infection.pdf. Accessed April 16, 2020.

Children's Hospital of Philadelphia, Shaw K, Plachter N, et al. 2018 June. Pathway for the Evaluation and Treatment of Children with Febrile UTI. Available from: https://www.chop.edu/clinical-pathway/urinary-tract-infection-uti-febrile-clinical-pathway. Accessed April 16, 2020.

Dell Children's Medical Center, Miner G, Hebner C, et al. 2017 May. First Febrile Urinary Tract Infection. Available from: https://www.dellchildrens.net/wp-content/uploads/sites/60/2019/08/DCMC-Urinary-Tract-Infection-Clinical-Pathway.pdf. Accessed April 16, 2020.

Doganis D, Mavrikou M, Delis D, et al. Timing of voiding cystourethrography in infants with first time urinary infection. *Pediatr Nephrol*. 2009;24(2): 319-322.

Keren R & Chan E. A meta-analysis of randomized, controlled trials comparing short- and long-course antibiotic therapy for urinary tract infections in children. *Pediatrics*. 2002;109(5): e70. doi: 10.1542/peds.109.5.e70.

Kowalsky RH, Rondini AC, Platt SL. The Case for Removing Race From the American Academy of Pediatrics Clinical Practice Guideline for Urinary Tract Infection in Infants and Young Children With Fever. *JAMA Pediatr*. 2020;174(3):229-230. doi:10.1001/jamapediatrics.2019.5242

Lavelle JM, Blackstone MM, Funari MK, et al. Two-Step Process for ED UTI Screening in Febrile Young Children: Reducing Catheterization Rates. *Pediatrics*. 2016;138(1): e20153023. doi: 10.1542/peds.2015-3023

Michael M, Hodson EM, Craig JC, et al. Short compared with standard duration of antibiotic treatment for urinary tract infection: a systematic review of randomised controlled trials. *Arch Dis Child*. 2002;87(2): 118-123. doi: 10.1136/adc.87.2.118

National Institute for Health and Care Excellence. *Urinary tract infection in under 16s: diagnosis and management (CG54).* 2018. Available from https://www.nice.org.uk/guidance/cg54#. Accessed April 16, 2020.

National Institute of Allergy and Infectious Diseases. Short Course Therapy for Urinary Tract Infections in Children. Identification No. NCT01595529. 2012. Available from https://clinicaltrials.gov/ct2/show/NCT01595529. Accessed April 16, 2020.

Pauchard JY, Chehade H, Kies CZ, et al. Avoidance of voiding cystourethrography in infants younger than 3 months with Escherichia coli urinary tract infection and normal renal ultrasound. *Arch Dis Child*. 2017;102(9): 804-808. doi: 10.1136/archdischild-2016-311587.

Ristola MT, Loyttyniemi E, Hurme T. Factors associated with abnormal imaging and infection recurrence after a first febrile urinary tract infection in children. *Eur J Pediatr Surg*. 2017;27(2): 142-149. doi: 10.1055/s-0036-1572418.

Saltychev M, Ristola MT, Laimi K. Accuracy of ultrasonography in predicting vesicoureteral reflux in children: A meta-analysis. *Scand J Urol*. 2016;50(4): 239-245. doi: 110.1080/21681805.2016.1194462

© 2018 Children's Hospital and Health System, Inc.

Seattle Children's Hospital, Taxier R, Austin E, et al. 2019 April. Urinary tract Infections (UTI) Pathway. Available from: https://www.seattlechildrens.org/pdf/UTI-pathway.pdf. Accessed April 16, 2020.

Shaikh N, Hoberman A, Hum SW, et al. Development and Validation of a Calculator for Estimating the Probability of Urinary Tract Infection in Young Febrile Children. *JAMA Pediatr*. 2018;172(6):550-556. doi:10.1001/jamapediatrics.2018.0217

Shaikh N, Morone NE, Bost JE, et al. Prevalence of Urinary Tract Infection in Childood: A Meta-Analysis. *The Pediatr Infect Dis J*. 2008;27(4): 302-309.

Shaikh N, Spingarn RB, Hum SW. Dimercaptosuccinic acid scan or ultrasound in screening for vesicoureteral reflux among children with urinary tract infections. *Cochrane Database Syst Rev*. 2016; 7. doi: 10.1002/14651858.CD010657.pub

Simren Y, Stokland E, Lagerstrand KM, et al. Ultrasound is an effective and noninvasive method of evaluating renal swelling in infants with their first urinary tract infection. *Acta Paediatr*. 2017;106(11): 1868-1874.

Subcommittee on Urinary Tract Infection. Reaffirmation of AAP Clinical Practice Guideline: The Diagnosis and Management of the Initial Urinary Tract Infection in Febrile Infants and young Children 2-24 Months of Age. *Pediatrics*. 2016;138(6), e20163026. doi: 10.1542/peds.2016-3026

Subcommittee on Urinary Tract Infection, Steering Committee on Quality Improvement and Management. Urinary Tract Infection: Clinical Practice Guideline for the Diagnosis and Management of the Initial UTI in Febrile Infants and Children 2 to 24 Months. *Pediatrics*. 2011;128(3): 595-610. doi: 10.1542/peds.2011-1330

Sutton AG, Chandler N, Roberts KB. Recent studies on the care of first febrile urinary tract infection in infants and children for the pediatric hospitalist. *Rev Recent Clin Trials*. 2017;12(4): 269-276. doi: 10.2174/1574887112666170816143639.

Vyas DA, Eisenstein LG, Jones DS. Hidden in Plain Sight - Reconsidering the Use of Race Correction in Clinical Algorithms. *N Engl J Med*. 2020;383(9):874-882. doi:10.1056/NEJMms2004740

Wang ME, Lee V, Greenhow TL, et al. Clinical response to discordant therapy in third-generation cephalosporin-resistant UTIs. *Pediatrics*. 2020;145(2): e20191608.

Westwood ME, Whiting PF, Cooper J, et al. (2005). Further investigation of confirmed urinary tract infection (UTI) in children under five years: a systematic review. *BMC Pediatr*. 2005;5(2). doi: 10.1186/1471-2431-5-2

© 2018 Children's Hospital and Health System, Inc.

Approved by the CW UTI Pathway Team on March 17, 2021

CW Urinary Tract infection (UTI) Pathway Team Members:

Shannon Baumer-Mouradian, MD | Emergency Medicine, Pathway Team Lead
Jonathan Ellison, MD | Urology, Pathway Consultant
Sri Chinta, MD | Emergency Medicine
Michelle Mitchell, MD | Infectious Disease
Sarah Bauer, MD | Hospital Medicine
Anika Nelson, MD | Hospital Medicine
Jessica De Valk, MD | Primary Care
Aaron Hanson, MD | Primary Care
Ben Landgraf, MD | Primary Care
Jennifer Miller, PA-C | Primary Care
Bob Rohloff, MD | Primary Care
Jaspreet Samra | Primary Care Project Specialist
Danielle Smith, MSN, RN, CNL | Primary Care, Clinical Practice Leader
Sadia Ansari, MD | Urgent Care
Ilana Cabrera, MSN, RN, CPNP | Urgent Care, Clinical Practice Leader
Karie Mantey, MD | Urgent Care
Laura Marusinec, MD | Urgent Care
Kristine Wake, MD | Urgent Care
Katie Ray, PharmD | Antimicrobial Stewardship Pharmacist
Glenn Bushee | PAI Analyst
Liz Witkowski, MLIS | Clinical Services Librarian
Ian Reineking | Clinical Informaticist
Lia Bradley, MSN, RN, CNL, FNP | Clinical Guidelines Coordinator

© 2018 Children's Hospital and Health System, Inc.
See Medical Disclaimer

Version History and Summary of Changes

- Version 1.0 (4/5/2021): Go-Live

© 2018 Children's Hospital and Health System, Inc.

Medical Disclaimer

Medicine is a dynamic science; as research and clinical experience enhance and inform the practice of medicine, changes in treatment protocols and drug therapies are required. The authors have checked with sources believed to be reliable in their effort to provide information that is complete and generally in accord with standards accepted at the time of publication. However, because of the possibility of human error and changes in medical science, neither the authors nor Children's Hospital and Health System, Inc. nor any other party involved in the preparation of this work warrant that the information contained in this work is in every respect accurate or complete, and they are not responsible for any errors in, omissions from, or results obtained from the use of this information. Readers are encouraged to confirm the information contained in this work with other sources.

© 2018 Children's Hospital and Health System, Inc.
