# Supplementary material for: Applying Quality Improvement Methodology to Standardize Pediatric Urinary Tract Infection Diagnosis and Management throughout a Healthcare System
Source: Pediatr Qual Saf. 2024 Aug 21;9(5):e756. doi: 10.1097/pq9.0000000000000756 (PMC11338250; doi:10.1097/pq9.0000000000000756)
Supplement: Supplementary file 2 [file pqs-9-e756-s002.pdf]

Supplemental Data – ICD-10 Diagnosis Codes Used in Data Analysis for Suspected UTI, Exclusion Parameters, and Return Visits

Suspected UTI Diagnosis Codes:

Dysuria	R30.0
Fever in pediatric patient	R50.9
Urinary frequency	R35.0
Generalized abdominal pain	R10.84
Unspecified abdominal pain	R10.9
Acute cystitis without hematuria	N30.00
Acute cystitis with hematuria	N30.01
Urinary tract infection without hematuria, site unspecified	N39.0
Lower abdominal pain	R10.30
Urinary incontinence, unspecified type	R32
Right lower quadrant abdominal pain	R10.31
Hematuria, unspecified type	R31.9
Painful urination	R30.9
Pyelonephritis	N12
Periumbilical abdominal pain	R10.33
Cystitis	N30.90
Retention of urine, unspecified	R33.9
Urinary tract infection with hematuria, site unspecified	N39.0, R31.9
Urinary urgency	R39.15
Left lower quadrant abdominal pain	R10.32
Gross hematuria	R31.0
Foul smelling urine	R82.90
Acute pyelonephritis	N10
Chronic abdominal pain	R10.9, G89.29
Fussy infant	R68.12
Cystitis with hematuria	N30.91
Fussiness in child > 1 year old	R45.89
Bacteriuria	R82.71
Frequency of urination and polyuria	R35.0, R35.89
Other retention of urine	R33.8
Combined abdominal pain, vomiting, and diarrhea	R10.9, R19.7, R11.10
Pyuria	R82.81
Abdominal pain, vomiting, and diarrhea	R10.9, R11.10, R19.7
Upper abdominal pain, unspecified	R10.10
History of gross hematuria	Z87.898
History of pyelonephritis	Z87.448
E. coli UTI	N39.0, B96.20
Hematuria with proteinuria	R31.9, R80.9
Abdominal pain with vomiting	R10.9, R11.10
UTI due to Klebsiella species	N39.0, B96.89
Nonobstructive reflux-associated chronic pyelonephritis	N11.0
Bilateral groin pain	R10.31, R10.32
Frequent urination at night	R35.1
Urinary tract bacterial infections	N39.0, A49.9

Septic shock due to urinary tract infection	A41.9, R65.21, N39.0
Urinary tract infection due to extended-spectrum beta lactamase (ESBL) producing Escherichia coli	N39.0, B96.29, Z16.12
Idiopathic hematuria, unspecified whether glomerular morphologic changes present	N02.9
Hypercalciuria	R82.994
Chronic RLQ pain	R10.31, G89.29
Tubulointerstitial nephritis and uveitis	N12, H22
Chronic generalized abdominal pain	R10.84, G89.29
Hypercalciuria	A41.9, N39.0
Urinary tract infection associated with cystostomy catheter, initial encounter	T83.510A, N39.0
Flank pain with history of urolithiasis	R10.9, Z87.442
Fever with sore throat	R50.9, J02.9
Dysuria	N39.0, B96.4
Dysuria	J98.9, R50.9
Sepsis due to gram-negative UTI	A41.50, N39.0
Bowel and bladder incontinence	R32, R15.9
Chronic bilateral lower abdominal pain	R10.31, G89.29, R10.32
Pain due to retention of urine	R33.9, R52
Fever in pediatric patient	N39.0, B96.5
Pyelonephritis due to Escherichia coli	N12, B96.20
Traumatic injury of abdomen with gross hematuria	S39.91XA, R31.0
Enterococcus UTI	N39.0, B95.2
Postoperative abdominal pain	R10.9, G89.18
Bacteriuria with pyuria	R82.71, R82.81

Exclusion Diagnosis Codes:

Neurogenic bladder	N31.9
Diaper Candidiasis	B37.2
Candida vaginitis	B37.3
Yeast Infection	B37.9
Diaper Dermatitis	L22
Vesicoureteral reflux w/ scarring	N13.70
vesicoureteral reflux without nephropathy	N13.71
Nephrolithiasis	N 20
bladder calculus	N 21
neuropathic bladder	N 31
retention of urine	N 33
Epididymitis	N45.1
Balanitis	N48.1
Spina Bifida	Q05
Epispadias	Q64.0
Exstrophy of urinary bladder	Q64.1
Congenital posterior urethral valves	Q64.2
Other atresia and stenosis of urethra and bladder neck	Q64.3
Congenital urethrorectal fistula	Q64.73
history of urinary bladder augmentation	Z96.0
Other inflammation of vagina and vulva	N76

Acute vaginitis	N76.0
Subacute and chronic vaginitis	N76.1
Acute vulvitis	N76.2
Subacute and chronic vulvitis	N76.3
Abscess of vulva	N76.4
Ulceration of vagina	N76.5
Ulceration of vulva	N76.6
Other specified inflammation of vagina and vulva	N76.8
Mucositis (ulcerative) of vagina and vulva	N76.81
Other specified inflammation of vagina and vulva	N76.89

Return Visit UTI Diagnosis Codes:

Acute cystitis	N30.0
Acute cystitis without hematuria	N30.00
Acute cystitis with hematuria	N30.01
Cystitis, unspecified without hematuria	N30.90
Cystitis, unspecified with hematuria	N30.91
Urinary tract infection, site not specified	N39.0
Acute pyelonephritis	N10
