# Supplementary figures and images for: Applying Quality Improvement Methodology to Standardize Pediatric Urinary Tract Infection Diagnosis and Management throughout a Healthcare System
Source: Pediatr Qual Saf. 2024 Aug 21;9(5):e756. doi: 10.1097/pq9.0000000000000756 (PMC11338250; doi:10.1097/pq9.0000000000000756)

## System Wide 14day Return Visits for UTI, P Chart

Percent 14d Return for UTI

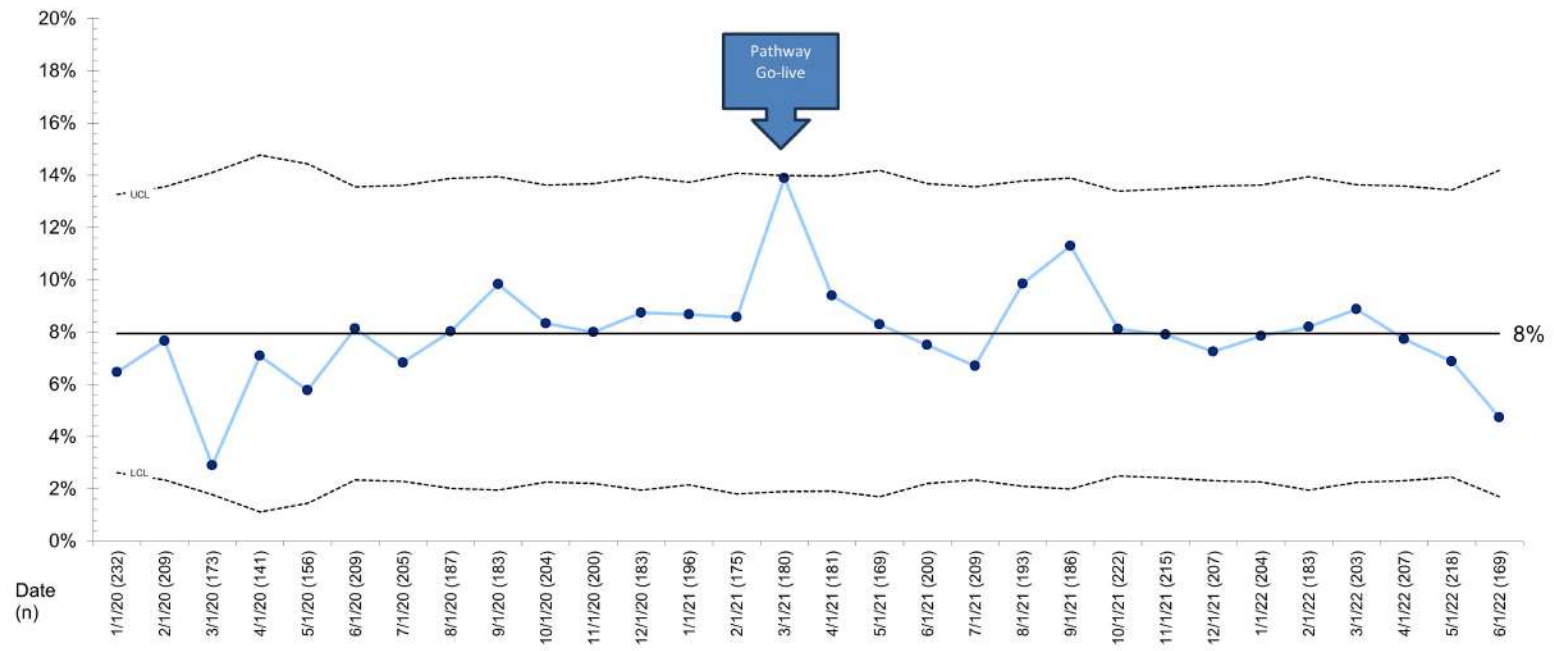

Supplement: Supplementary file 3 [file pqs-9-e756-s003.pdf]
